# The Development of Sustainable Polyethylene Terephthalate Glycol-Based (PETG) Blends for Additive Manufacturing Processing—The Use of Multilayered Foil Waste as the Blend Component

**DOI:** 10.3390/ma17051083

**Published:** 2024-02-27

**Authors:** Mikołaj Garwacki, Igor Cudnik, Damian Dziadowiec, Piotr Szymczak, Jacek Andrzejewski

**Affiliations:** 1 Faculty of Materials Engineering and Technical Physics, Poznan University of Technology, Piotrowo 3 Str, 60-965 Poznan, Poland; mikolaj.garwacki@student.put.poznan.pl (M.G.); igor.cudnik@student.put.poznan.pl (I.C.); 2 Institute of Materials Technology, Faculty of Mechanical Engineering, Poznan University of Technology, Piotrowo 3 Str, 61-138 Poznan, Poland; damian.dziadowiec@doctorate.put.poznan.pl (D.D.); piotr.szymczak@doctorate.put.poznan.pl (P.S.); 3 Eurocast Sp. z o.o., Wejherowska 9 Str, 84-220 Strzebielino, Poland

**Keywords:** additive manufacturing, foil waste, recycling, poly(ethylene terephthalate), polymer blends, mechanical performance, thermal resistance

## Abstract

The polymer foil industry is one of the leading producers of plastic waste. The development of new recycling methods for packaging products is one of the biggest demands in today’s engineering. The subject of this research was the melt processing of multilayered PET-based foil waste with PETG copolymer. The resulting blends were intended for additive manufacturing processing using the fused deposition modeling (FDM) method. In order to improve the properties of the developed materials, the blends compounding procedure was conducted with the addition of a reactive chain extender (CE) and elastomeric copolymer used as an impact modifier (IM). The samples were manufactured using the 3D printing technique and, for comparison, using the traditional injection molding method. The obtained samples were subjected to a detailed characterization procedure, including mechanical performance evaluation, thermal analysis, and rheological measurements. This research confirms that PET-based film waste can be successfully used for the production of filament, and for most samples, the FDM printing process can be conducted without any difficulties. Unfortunately, the unmodified blends are characterized by brittleness, which makes it necessary to use an elastomer additive (IM). The presence of a semicrystalline PET phase improves the thermal resistance of the prepared blends; however, an annealing procedure is required for this purpose.

## 1. Introduction

Plastic wastage is one of the emerging problems that humanity has to face [1,2,3]. Solid wastage is an enormous pollution problem, though foils are one of the worst. Despite microplastic formation, their low weight is also leading to collection problems—bags and other foil-made items are prone to be taken off by the wind from garbage, but also from farms, when are used as crop coverage, etc.

Among all polymeric materials, the largest share in global production is held by materials from the polyolefin group, mainly PE and PP; followed by thermoplastic polyesters, mainly PET; and styrene-based plastics. A large part of these materials are difficult to manage due to their multicomponent nature [4,5]. These issues could be resolved by increasing the number of foil re-usage processes, one of which is proposed in this article—a blend of filament for use in the FDM process, as this method uses thermoplastic materials with PET-based foils. The FDM (fused deposition modeling) method is a rapidly developing 3D printing technique that is cheap and easy to reproduce due to the many open-source printing systems. The big advantage of the FDM method is the easy and well-known filament-making process; thus, this work can be reproduced even without specialized processing equipment. The concept itself also allows for easy scaling-up of the printing process, which allows the production of middle and large-sized products, or even buildings when cement-based materials are used [6].

Most PET-based products are bottles and later textiles, which are 47% and 33% of European Union PET demand, respectively. The least in-demand products are PET packaging such as trays, flexibles, and strappings, and they are 13, 2 and 5% of demand. Bottles are the most recyclable products for now, as they are often captured from deposit return systems, and their infrastructure is developed, which is different from trays/flexibles, as well as textiles. This creates a 50% recycling rate for PET bottles and a 35% rate for PET packaging products [7]. Dziadowiec et al. [8] have shown that, nowadays, film production customarily employs two primary technologies—casting technology and blow molding technology. As opposed to blown film fabrication, cast film extrusion demonstrates enhanced operational effectiveness and provides superior uniformity in terms of film thickness.

According to Soares et al. [5], multi-layered packages (MMP) are demanding due to sorting, separating, identification, and technological issues; thus, such MMP products are usually sorted as another type of plastic and are usually used as plant power fuel, or, in low-income countries, MMP products are disposed of in dumpsites or land fields, which lead to pollution generation. Nowadays, there are several advanced methods to recycle MMP-based products [4,9], including new enzyme usage technologies to ensure the depolymerization process. Such substances secreted by some microorganisms might help degrade PET to its monomers [5]. Popular ways to recycle polymer composite materials are pyrolysis, hydrolysis, energy recovery, and compression/injection molding. Later, recycling by extrusion with compatibilizer blending was described, and reprocessing and compounding approaches were introduced and became more widely used. To sum up, these processes can be divided into two strategies: first, the compounds are separated, either by a dissolution-reprecipitation technique or by delamination of the multilayer. There are several ways to obtain delamination: physically (e.g., selective dissolution), chemically (e.g., reactive removal of an interlayer), or mechanically. On the other hand, the second strategy comprises the joint processing of the constituents of the multilayer packaging, though the use of additives is often necessary [4,10]. In the developed approach, switchable hydrophilicity solvents (SHSs) are used as sustainable chemicals to break the chemical and mechanical bonds between MFPW layers, thus allowing the separating of all the layers individually and achieving a recycling rate > 99% while making the reprocessing of extracted polymers much simpler [9]. Reactive extrusion is one of the most promising recycling strategies, allowing not only the mechanical creation of the blends but also the use of chemical compounds, for example, to enhance cross-linking. Przybysz-Romatowska et al. reviewed this strategy in various biopolymers in the presence of free-radical-initiators and presented positive results [11]. Candal et al. [12] have researched this method in the case of post-consumer recycled PET from opaque milk bottles produced as a blend of PET with TiO_2_ nanoparticles used in UHT (Ultra-high Temperature)-treated milk. Such packaging processes are replacing high-density polyethylene bottles. Other reports confirm the effectiveness of reactive processing for other PET-based blends [13,14,15,16,17].

Taking into account the growing market demand for various types of filaments, it is worth noting that, in addition to the development of advanced materials such as reinforced composites [18,19], conductive materials [20,21], or polymers for specialized applications [22,23,24], unmodified varieties of polymers still dominate the markets. Their growing demand is mainly related to the increasing number of non-professional and hobby users; additionally, the number of available large-size technologies is increasing [25,26,27,28]. For these cases, where the size of the prepared products requires the use of several kilograms of material, the important factors are the price of the material and its processability, particularly the intensity of the material flow through the extruder nozzle. Currently conducted research indicates the potential of using polyolefin waste [29,30], which could significantly reduce the price of processed materials. However, in the context of processability and extrusion process yield, the use of PET waste has even greater commercialization potential [31,32].

For the concept proposed in this article, the main advantage is the lack of process by-products; foil waste was introduced into the blending procedure without additional purification or chemical treatment. Filament production is a process that does not generate any by-products. The developed method may be a way of managing waste that is difficult to reuse in the production of other types of foil for this reason. Obvious issues related to the LCA analysis of FDM filament products, estimation of the level of energy consumption, and greenhouse gas emissions might be a broad topic for discussion [33,34,35,36], but such a complex analysis was not the main topic of this research work. A detailed analysis could obviously help in accurately assessing the impact of the addition of foil to the tested polymer materials, but, from the point of view of the end user, the more important information is the functional properties that can be obtained for the developed materials [32]. Hence, the main goal of the presented research was a general assessment of the properties of modified materials and an indication of possible directions for further work in the field of potential commercialization.

The annealing procedure that was additionally applied after the printing process aimed to improve the thermal resistance. For thermoplastic polyester-based materials, where the cold crystallization phenomenon is present for many commercially available polymers, this procedure has been utilized very often, especially for PLA [37,38,39] and PET [40,41,42], which significantly improves the thermomechanical properties.

The developed concept of polymer waste management using a 3D printing method is a relatively new idea and results mainly from the growing interest in new additive manufacturing techniques [43,44,45,46,47]. In last few years, some similar research topics concerned the utilization of FDM printing process wastes, which mainly concerns the modification of various types of PLA filaments [48,49,50]. Some works also cover various ways of using PET waste [51,52]. The presented study is focused on the development of new types of materials devoted to FDM-method processing. The aim of this work was to develop materials with the best possible functional properties; therefore, the content of foil waste was limited to approximately 40–50%, while the rest of the polymer composition itself was prepared from the PETG copolymer dedicated to FDM processing. In order to improve the mechanical properties and obtain better phase compatibility of the polymer mixture, the materials were processed using the reactive extrusion method; additionally, a POE-g-GMA elastomer phase was used as the impact modifier (IM). The prepared materials were subjected to a detailed analysis of their mechanical, thermal, and rheological properties. Some of the work results included a comparative analysis performed for 3D printed and injection molded samples.

## 2. Experimental Section

### 2.1. Materials

During this study, we used poly(ethylene terephthalate)-glycol copolymer (PETG) as the main component of the prepared blends. The used type of PETG material was commercially available filament from Devils Design company. Materials were supplied in the form of unmodified, transparent resin. Before processing, the filament spool was ground into the form of pellets. 

The second component of the prepared blends was made from PET-based foil waste. For the purpose of this study, we used three types of laminated foils. Materials of this type have already been used by us in previous research on foil applications [53]. Foil 1, marked as PET/PE, was prepared during the lamination of APET foil with a layer of polyethylene (LLDPE). Foil 2, marked as PET/EVOH, was made from APET and laminated with a PE layer and ethylene-vinyl alcohol layer (EVOH). Foil 3, marked as PET/MET, was prepared from the laminated APET/PE material additionally covered with an aluminum layer (≈10 nm); for aesthetic purposes, the foil was colored with yellow dye. All three types of foil materials were supplied in the form of flakes; in order to homogenize their properties, the foils were initially melt-blended using a single screw extruder. The process was conducted at 265 °C, where the obtained materials were pelletized and stored for the next stage of processing.

For the purpose of modification, we used a reactive chain extender (CE) as a blend compatibilizer and an elastomeric copolymer as an impact modifier (IM). The CE compound was an epoxy-based copolymer SAG-008 from the Fine Blend company. This CE consisted of styrene-acrylonitrile-glycidyl methacrylate random copolymer (SAN-g-GMA). The IM compound was a polyolefin-based copolymer functionalized with epoxy groups (POE-g-GMA); the type of elastomer used was SOG-03 (company Fine-blend, Shanghai, China).

### 2.2. Sample Preparation

The main preparation process was a melt blending procedure conducted using a twin-screw extruder. For this purpose, we used a Zamak EH16.2D machine (Zamak Mercator, Skawina, Poland), where the extruder barrel profile was set as follows: 250-250-260-270-270-260-260-260-250 °C, the screw speed was 130 rpm, and the extrusion output was set to around 2 kg/h. The material was supplied volumetrically. The obtained extrudate was cooled down and pelletized. Before processing, the PETG and recycled PET foil pellets were dried using a cabinet oven (70 °C/12 h). The full list of samples, together with the formulations, is listed in Table 1. The pellets prepared during the melt blending process were used for the sample preparation. For the purpose of the discussed study, the main goal was to prepare 3D-printed parts, which is why the prepared materials were processed into filaments and shaped using the FDM technique. The reference samples were prepared by injection molding.

**Figure 1:**
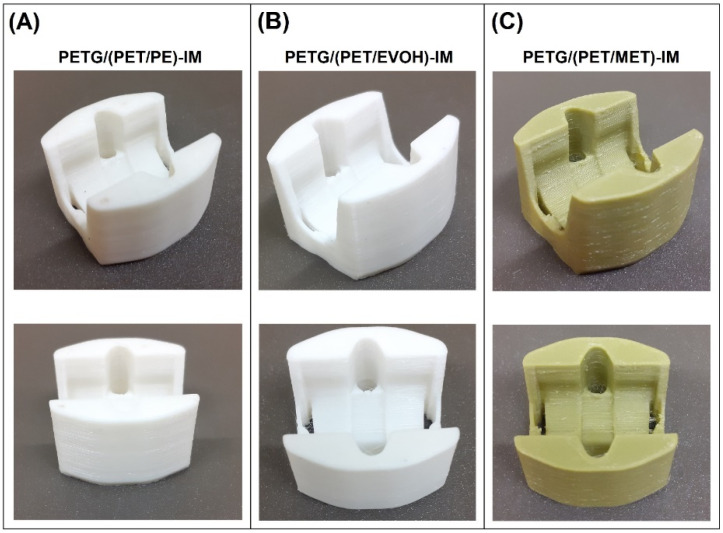
The appearance of the FDM printed samples:. The presented parts can be used for mounting sliding bearing elements.

The filament extrusion procedure was conducted on a single screw extruder, model Metalchem W25-30D machine (IMPiB, Torun, Poland). The machine die-head was equipped with a 2 mm diameter nozzle. The extrusion process was conducted at 245 °C, measured at the die. The screw speed was set to 20 rpm, which enabled the process efficiency to be around 1.5 kg/h. The extruded melt was transferred to the dry conveyor and collected on the spool using the filament winder. The 3D printing procedure was conducted on a benchtop FDM machine, model Prusa MK3S (PrusaResearch, Prague, Czech Republic). The machine was equipped with a 0.6 mm brass nozzle. The printing parameters were constant for all materials. The nozzle temperature was set to 245 °C; the platform temperature was 90 °C. The standardized samples were printed as solid models (100% infill), the layer height was set to 0.2 mm, and the printed models were prepared with two outer (shell) layers. The printing speed for infill layers was 60 mm/s, while for the shell layer it was 30 mm/s. In order to evaluate the printing accuracy, we prepared the part with more complex geometry in the form of mounting to the sliding bearing. The appearance of the part geometry for selected materials is presented in Figure 1.

The injection molding process was conducted using a standard hydraulic press, Engel Victory 550 (Engel GmbH, Schwertberg, Austria). The injection temperature was set to 260 °C, while the mold temperature was 30 °C. The injection/holding pressure was 900/450 bar. The holding/cooling time was 10/30 s. Before testing, the 3D printed and molded specimens were conditioned for at least 48 h in sealed bags.

### 2.3. Characterization

The mechanical performance of the prepared samples was measured using the static tensile technique and Charpy impact resistance measurements. For tensile testing, we used a Zwick/Roell Z010 universal testing machine (load cell 10 kN) (ZwickRoell, Ulm, Germany). The tests were conducted according to ISO 527 standard [54]. The measurements were conducted at a test rate of 5 mm/min. The impact resistance measurements were conducted using a Charpy hammer, model Zwick/Roell HIT 15. The machine was equipped with a 5 J energy pendulum. The notching procedure was made after sample printing/molding. The whole testing procedure was conducted according to ISO 179 standard [55].

The thermal properties of the manufactured samples were determined using the DSC method. For our study, we used the standard heat flux apparatus DSC F1 204 Phoenix (from Netzsch Geratebau, Selb, Germany). The tests were conducted in 25 mL aluminum crucibles, where the average weight of the sample was 5 mg. The test program consisted of three main stages: 1st heating, cooling, and 2nd heating, where the oven chamber was purged with a protective nitrogen atmosphere (40 mL/min) during the whole procedure. The heating/cooling was set to 10 °C/min, while the temperature range was 20–300 °C. Samples were cut from the 3D-printed specimens and corresponding annealed samples. 

The viscoelastic properties of the prepared samples were investigated using the DMTA analysis method. For this purpose, we used a Netzsch DMA 242E Artemis apparatus (NETZSCH Analyzing & Testing, Selb, Germany). The machine was equipped with dual cantilever clamps. The measurements were performed from 25 °C to 150 °C, where the maximum temperature limit was estimated during preliminary tests to limit excessive softening of the sample. The heating rate was 2 °C/min, the amplitude frequency was set to 1 Hz, and the amplitude range was 15 μm. The collected data were presented in the form of storage modulus and tan δ plots. The tests were conducted on FDM printed samples with the dimensions 10 mm × 10 mm × 60 mm.

The thermomechanical properties were evaluated using the heat deflection method, where tests were conducted according to ISO 75 standard [56]. The tests were carried out using a Testlab RV300C HDT/Vicat machine (Testlab, Warszawa, Poland). During the test, the samples were immersed in the oil chamber. The applied load was 1.8 MPa; the heating rate was set to 120 °C/h.

A scanning electron microscope (SEM) type MIRA3 (Tescan, Brno, Czech Republic) was used for the purpose of structural analysis. Before scanning, the sample was coated with a thin layer of carbon (≈20 nm) using a Jeol JEE 4B vacuum evaporator (JEOL, Tokyo, Japan).

The rheological analysis of the prepared samples was made using the rotational rheometer. For this purpose, we used an Anton Paar MCR301 apparatus (Anton Paar, Graz, Austria). The tests were conducted using 25 mm diameter discs prepared by compression molding from the pelletized blends at 250 °C. The measurements were conducted at 250 °C, which was close to the manufacturing temperature range. The machine was equipped with a 25 mm diameter plate–plate system, working at a 0.5 mm distance gap. The frequency range was set to 0.1–100 rad/s, and the amplitude of 0.5% was determined during the preliminary measurements (amplitude sweep test). The validation tests in the form of melt flow rate measurements (MFR) were conducted using a Dynisco D4004 melt flow indexer (Dynisco, Heilbronn, Germany). The tests were performed at 250 °C, with an applied load of 2.16 kg. The measurements were conducted according to ISO 1133 [57]; however, due to a lack of recommendations for PETG/PET-based materials, we modified the test parameters to reflect the processing conditions.

## 3. Results and Discussion

### 3.1. Mechanical Performance—Static Tensile Tests and Impact Resistance Evaluation

**Figure 2:**
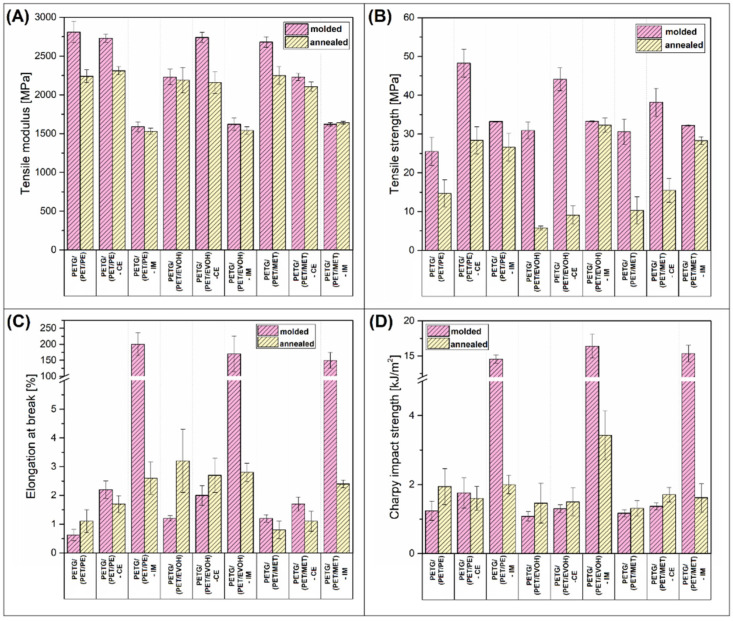
The results of the mechanical properties evaluation for injection molded samples and molded/annealed specimens: (**A**) tensile modulus; (**B**) tensile strength; (**C**) elongation at break; (**D**) impact strength.

**Figure 3:**
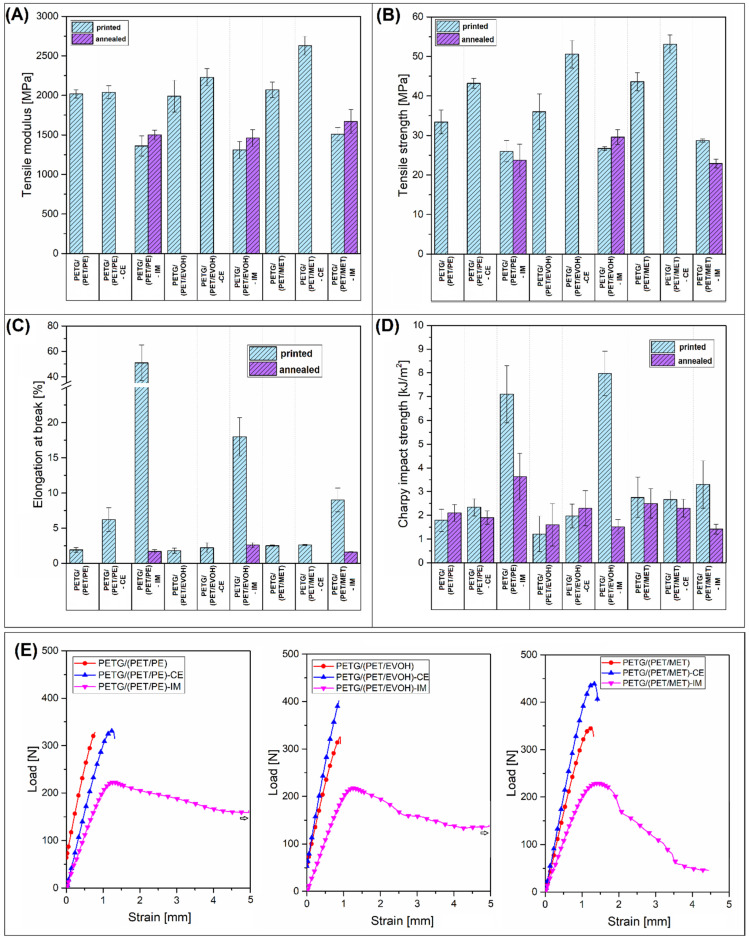
The results of the mechanical properties evaluation for FDM printed samples and printed/annealed specimens: (**A**) tensile modulus; (**B**) tensile strength; (**C**) elongation at break; (**D**) impact strength; (**E**) tensile test curves of the prepared (printed) materials.

The properties of the manufactured specimens were investigated using static tensile and Charpy impact testing. The results are divided into two figures, where mechanical characteristics for injection molded and 3D printed materials are presented in Figure 2 and Figure 3, respectively.

The plots collect the results for untreated materials (molded/printed) and annealed samples. It is worth noting that some of the FDM printed specimens were too brittle to mount them in the machine clamps, which is why the tensile properties are missing. Additionally, the tensile test curves are collected for the 3D printed samples (Figure 3E).

It is a common trend that, in a direct comparison between molded and printed specimens, the properties are mostly in favor of the injection molding method. For the investigated samples, this is mostly true. The analysis of the tensile properties revealed some minor differences in modulus, where the results for the molded samples are usually slightly higher than the stiffness of 3D printed materials; however, the overall trends are similar. The tensile modulus results for the untreated blends and CE-modified samples are mostly the same, with some small random changes. The addition of the IM compound leads to a large reduction in stiffness, which is a typical behavior after the addition of an elastomeric phase. Interestingly, for the molded samples, the annealing procedure leads to a small reduction in tensile modulus, while, for most studies, the annealing procedure increases the stiffness of polyesters [42,58]. It seems that for the prepared blends the additional heat treatment leads to high brittleness, which translates into tensile modulus/strength deterioration. The strength value is the most affected factor, since for most of the molded samples the recorded decrease was more than 50% in comparison to the reference results. For the 3D printed samples, the strength reduction was even higher since the brittleness of the specimens made it impossible to conduct the tensile test. Interestingly, when comparing the tensile strength/modulus of IM-modified materials, the properties of molded and printed samples are very similar; moreover, the annealing procedure does not lead to extensive brittleness. 

This favorable effect of the IM addition is more evident when analyzing the elongation at break and impact strength results. The maximum strain for unmodified molded samples did not reach 3%, which confirmed that even the reactive compatibilization was not an effective procedure for brittleness reduction. Interestingly, the results for unmodified and heat-treated (annealed) samples were slightly similar, which means that the morphology of the PET crystalline phase is not a deciding factor influencing the deformation mechanism. The addition of POE-g-GMA elastomer led to a large increase in material ductility, since the elongation at break for all of the molded materials was in the range of 150–200%. The same differences were observed for impact strength values, where the results for toughened samples were significantly better. Similar to the strain at break values, the Charpy test results revealed that for most of the materials the impact resistance was close to the results of brittle types of thermoplastics, such as poly(lactic acid)–PLA, or polystyrene–PS [59,60,61,62], which translates to being 2 kJ/m^2^ or lower. A visibly more favorable deformation mechanism characterizes the IM-modified materials since most of the samples reached 15 kJ/m^2^ in Charpy tests. The same trends apply to the FDM printed materials; however, overall, the results for particular types of materials were always better for injection molded samples. The best results of the elongation at break measurements were obtained for the PETG/(PET/PE)-IM sample, where the mean values were close to 50%. For the PETG/(PET/EVOH)-IM and PETG/(PET/MET)-IM samples, the recorded strain was 16% and 8%, respectively, which confirmed a large advantage of the molding process. The improved toughness of IM-modified materials is easy to determine when comparing tensile test plots. The curve comparison from Figure 3E reveals the visible trend for all the blend groups. For all samples with the addition of IM elastomer, the ultimate elongation and the strain at the yield value point are the highest, which clearly suggests higher flexibility for the given samples. For the remaining samples, the inflection point is achieved only for selected materials, which indicates a brittle fracture mechanism. 

The comparison of the impact strength values revealed slightly better results. The impact resistance for PETG/(PET/PE)-IM and PETG/(PET/EVOH)-IM materials was 7 kJ/m^2^ and 8 kJ/m^2^, respectively. However, for PETG/(PET/MET), it drops again to 3.5 kJ/m^2^. The observed results strongly correlate with the previous studies, where the direct comparison between the molded and printed samples revealed that for the FDM printed samples the strain values are strongly reduced by the structure discontinuities, while the impact strength can even be similar to the results obtained for injection molded samples [63]. Unfortunately, the additional thermal treatment (annealing) led to a large deterioration in sample ductility. The poor results are not surprising for samples without the addition of IM. Still, even when using an elastomer addition, the results return to the reference values recorded for unmodified materials. This behavior clearly indicates a lack of perspective for obtaining good mechanical properties for annealed samples. At the same time, this indicates the need to find another way to improve thermal resistance.

As a summary of some of this research on the mechanical properties of the developed works, it is worth analyzing the results of this work in the context of the previous research where FDM printing was used as a technology for shaping polymer waste. Table 2 is collecting the basic mechanical characteristics like E modulus, tensile strength and elongation at break. We considered only polyester-based materials that were ultimately produced by the FDM printing process, excluding works where waste from the 3D printing process was used to produce foil or injection molded products. Mechanical properties were determined only for selected works in this field, which further shortened the discussed list. The existing studies mainly related to PET and PLA recycling and include some interesting results. The topic of a few papers was related to the processing of polymer blends containing polyolefins (PP and PE), a concept that is very close to the subject of the currently described research [64,65,66]. However, it is worth noting that, unlike works devoted to research on injection molded or extruded materials, the discrepancy in the results for research in the field of 3D printing technology is very large, and covers also the reinforced materials [67]. For example, the study by Agbakoba et al. [68] revealed that the E modulus of PLA samples after reprocessing can reach over 5 GPa, without the reinforcing filler. This result seems doubtful, considering the results of other works [69]. It is, therefore, worth adding that any comparative analysis within the FDM technique is very dependent on the process conditions, primarily the method of orientation of the material in the production process, which may significantly affect the uniformity of the results. Unfortunately, unlike tests for injected samples, there are no clear guidelines regarding the measurement methodology for additively manufactured samples.

### 3.2. Thermal Properties of Prepared Materials—Differential Scanning Calorimetry Measurements (DSC)

**Figure 4:**
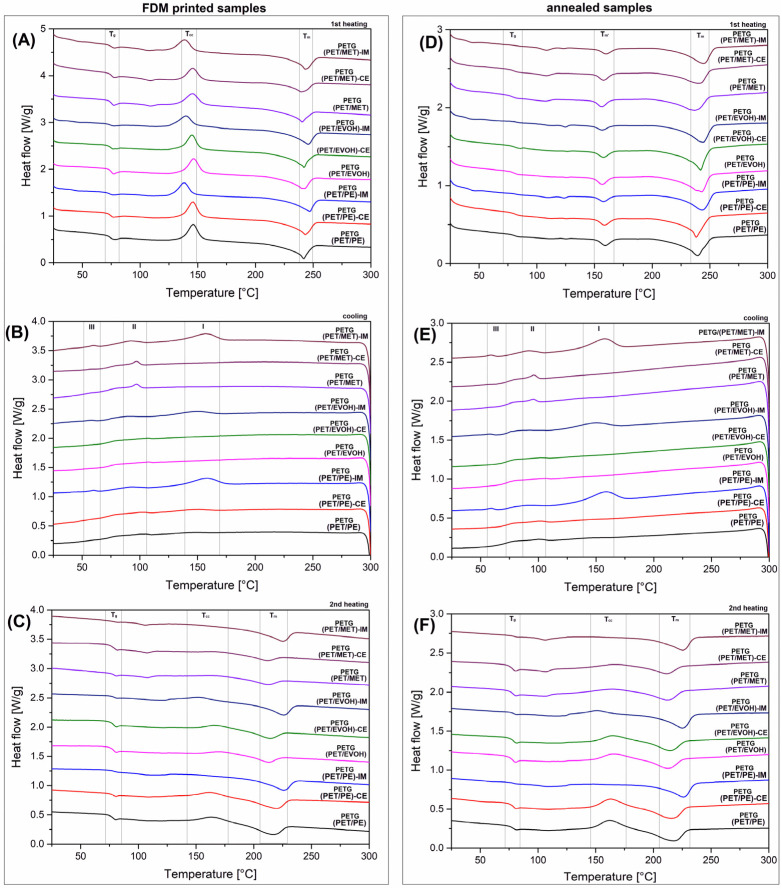
The DSC signals for all of the prepared blends were obtained from the subsequent stages of the test, 1st heating/cooling/2nd heating. The results are collected for FDM printed samples (**A**–**C**) and printed/annealed samples (**D**–**F**).

The results of the DSC analysis are presented in the form of a thermogram comparison. The chart compilation from Figure 4 shows the 1st heating, cooling, and 2nd heating thermograms for FDM printed samples and annealed specimens.

A brief analysis of the 1st heating plots revealed that the PET component phase transitions dominate the DSC signals. For all of the investigated materials, there are three characteristic areas of the DSC thermogram. The first one, recorded at around 75 °C, is related to the glass transition region (T_g_). For the prepared materials, the glass transition of the PETG component and PET from the foil waste overlap, which results in a single inflection point. For most of the investigated samples, the T_g_ point was recorded from 70 °C to 75 °C, which means that the blend composition did not influence the glass transition phenomenon. The second characteristic area was the cold crystallization region (T_cc_). Since the PETG copolymer usually reveals fully amorphous characteristics, the cold crystallization phenomenon is strongly combined with the presence of the PET homopolymer. For most of the samples, the peak area ranged from 120 °C to 160 °C; however, there are some minor changes in peak maximum position. When analyzing the results for IM-modified samples, it is clear that the T_cc_ position is shifted to a lower temperature compared to other samples. For example, the peak for PETG/(PET/MET) sample was recorded at 145 °C; the same temperature applies to the PETG/(PET/MET)-CE sample. Meanwhile, for the elastomer-modified material PETG/(PET/MET)-IM, the T_cc_ position moved to 138 °C. The same behavior was noticed for the other groups of materials, which confirmed that the observed phenomenon was directly connected with the presence of the POE-g-GMA phase [53,70]. Since this type of elastomer is functionalized by glycidyl methacrylate groups, strong interfacial interactions are likely formed between the polyester matrix and the elastomer inclusion [71,72,73,74]. The elastomer inclusions can act as nucleation surfaces, which improves the kinetic of crystalline phase formation. The last of the noticed regions was connected with the melting of the PET phase. The characteristic endothermic peak (T_m_) was recorded at different temperatures from 240 °C to 247 °C; however, no visible trend indicated any correlation between material composition and T_m_ changes. Summarizing the 1st heating stage of the DSC measurements, it can be concluded that the presence of a PET homopolymer phase mostly influenced the thermal behavior of the prepared blends. The visible cold crystallization area confirmed that the conditions of the FDM printing process are not optimal for the formation of highly crystalline structures. The presence of a small T_cc_ shift for IM-modified materials suggests the possible nucleation effect; however, the scale of this phenomenon was relatively small and did not increase the crystallinity level. 

The cooling stage thermograms presented in Figure 4B reveal again the dominating amorphous characteristic of the prepared blends. For most of the materials, the plot appearance does not reveal any strong exothermic phase changes, which might suggest the formation of a PET crystalline phase. A small exception from this trend is recorded for IM-modified samples, where, for all samples with the addition of POE-g-GMA phase, the thermogram analysis revealed a small and flat crystallization peak indicating crystalline structure formation in the temperature range of 140–170 °C (area I). The small area under the curve and its flattened shape suggests that the kinetic of this phenomenon was relatively low, which means that the resulting crystalline phase content will be small. It is worth noting here that, for measurements performed using the DSC technique at a relatively low cooling speed (10 °C/min), the formation of the crystalline phase is facilitated compared to the thermal conditions during the filament cooling stage in the FDM printing process. This means that, for real cooling conditions, the slow kinetic of PET crystalline phase formation does not allow for the evolution of highly crystalline structures. Interestingly, for PETG/(PET/MET) samples, there was a noticeable exothermic peak recorded in the range of 85–105 °C (area II). The most evident example of that phenomenon was noticed for unmodified blends and samples with a chain extender addition, the PETG/(PET/MET) and PETG/(PET/MET)-CE samples, where a very clear peak maximum was recorded at 95 °C. The peak signal is more diffused for PETG/(PET/MET)-IM materials. In our opinion, the noticed differences are related to the presence of the PE phase. For unknown reasons at this stage of research, its presence can be noted only for this type of material. The last of the observed characteristic phase transitions is related to the glass transition of the main amorphous components (area III). The deflection of the DSC signal was recorded at around 65 °C, which is a typical value for PET or PETG components.

The 2nd heating stage results presented in Figure 4C confirm our conclusions since the appearance of the obtained plots is strongly different from the 1st heating stage observations. For most of the investigated materials, the T_cc_ peak position was shifted from around 145 °C to 160 °C. Simultaneously, the melting area peak T_m_ was reduced from 245 °C to around 220 °C. Interestingly, for the 2nd melting stage, the IM-modified blends were clearly distinguished from the rest of the samples. In this case, no clear T_cc_ peak can be observed, which might be attributed to the increased crystallinity of the sample, which was confirmed during the cooling stage. The second difference applies to the T_m_ peak reduction, where, for samples with the addition of POE-g-GMA, the melting point was moved to around 230 °C, which is closer to the T_m_ from the 1st heating stage. The reason for the large temperature shift was the formation of undeveloped crystalline structures, where lamella thickness was relatively low compared to the structure from the 1st heating stage. Since the formation of the crystalline phase was facilitated during the cooling stage of the DSC test, a large part of the amorphous phase was bounded by crystalline regions and an area called the rigid amorphous phase (RAP). This results in the inability to form a regular crystalline phase at the stage of cold crystallization, which, in turn, despite the higher content of the crystalline phase, leads to the formation of structures with lower thermal stability.

Table 3 is collecting the data from the DSC signal plots, the listed temperature values are reflecting the differences in appearance of mayor phase transitions like glass transition (T_g_), cold crystallization (T_cc_), melting temperature (T_m_) or crystallization temperature (T_c_). The recorded values in the form of the inflection point of the DSC curve or its peaks are mostly related to the transformation of the PETG/PET blend system.

The DSC analysis for printed/annealed samples is presented similarly to untreated materials (see Figure 4). A direct comparison of the 1st heating thermograms revealed large differences between the phase transitions of the PET phase. In contrast to the appearance of the FDM printed specimens, the PET phase reveals a typical highly amorphous macromolecular structure. The annealing procedure performed at 150 °C was aimed at increasing the crystallinity of the PET phase. For 3D printed samples, the initial heating (1st heating) revealed that the cold crystallization phenomenon appears at around 140 °C. The annealing procedure was performed in order to utilize this characteristic and improve the crystallinity level of the blends. The DSC plots for the annealed samples revealed that the cold crystallization peak disappeared for all of the tested samples. Instead of the T_cc_ peak, the thermograms were attributed with a new endothermic peak close to 160 °C. Further heating causes the main melting peak to appear around 240 °C. The obtained results suggest that the crystalline structure formed during the annealing was not uniform. Considering the only small increase in the melting enthalpy area at T_m_, it is clear that only part of the amorphous phase was forming stable crystalline lamellar structures. The rest of the PET chains were taking part in the formation of a less organized crystalline phase. The described behavior was already observed for PET-based materials [75,76]; however, the appearance of the biphasic crystalline structure was not discussed broadly. Interestingly, the referred study was focused on the materials annealed at 180 °C, which strongly correlates with the smaller melting peak observed on the DSC plot. The same conclusions appear for the presented study, where the annealing procedure was performed at 150 °C.

From a purely scientific point of view, the phenomena occurring during the formation of the phase structure of the developed materials could constitute a separate research topic. In particular, the kinetics of the formation of the crystalline phase. However, a certain limitation is the lack of precise characteristics and composition for the PET film component, which, basically, for each of the three types of film consists of at least two types of polymer, PET and PE, EVOH, or three-component systems. This is a major obstacle to advanced structural research such as crystallization kinetics measurements using the DSC or hot-stage microscopy methods. The detailed information on the composition of the material is of enormous importance in the interpretation of the results. An interesting example of research on doping PET with the addition of a small amount of polyolefins is the work of Vaucher et al. [64], where the addition of high-density polyethylene in the PET filament was analyzed. In the case of these tests, a more detailed phase analysis would be possible, but, similarly to those discussed in this work, the authors focused on the analysis of mechanical properties. Examples of other works where polymer mixtures of the PET/polyolefin type were used during 3D printing indicate that the interpretation of results for polymer systems with undefined composition is extremely difficult and subject to a lot of assumptions [65,66,77]. 

Additionally, an important factor worth taking into account when analyzing the crystallization process is the correlation of this process in simulated conditions, as in DSC measurements, and real conditions, as in the injection molding and FDM printing process [78,79,80,81]. The Standard DSC tests are not able to reflect the thermal conditions of the real processes even for relatively non-dynamic changes such as in 3D printing. For the research discussed here, the most problematic aspect of DSC measurements is the cooling stage, where it would be desirable to obtain a similar level of cooling. Such conditions are possible when using a FlashDSC device (Mettler Toledo, Columbus, OH, USA). Unfortunately, this technique is very expensive and requires a complicated sample preparation methodology. For the discussed research, where the thermal conditions of the processes are not an analyzed variable, crystallization kinetics studies would not provide significant information on structure–property correlations.

Summarizing the analysis of the thermal properties measured using the DSC method, it is worth noting that the cooling and 2nd heating stage of the tests revealed that, for annealed samples, the appearance of the DSC plots is very similar to the results obtained for untreated (FDM printed) specimens. That fact proves that the processing procedure was the main factor influencing the phase transition differences recorded during the 1st heating stage. The described analysis could be more detailed if the crystallinity level were calculated. Unfortunately, due to the complex nature of the obtained DSC signals, it was impossible to collect the necessary data. Additionally, the exact fraction of the PET phase was not possible to estimate since the used foil components were not characterized by the producer. The content of the LDPE and EVOH compounds may vary, which makes the crystallinity calculations imprecise. Therefore, the data collected in Table 3 should not be treated as precise. 

### 3.3. Thermomechanical Properties—DMTA/HDT Measurements

**Figure 5:**
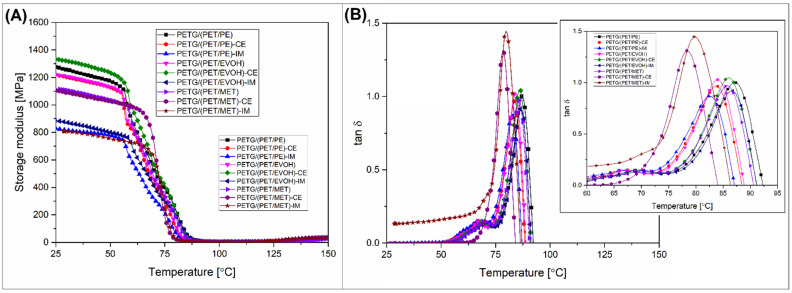
The DMTA results for FDM printed materials: (**A**) storage modulus; (**B**) tan δ.

**Figure 6:**
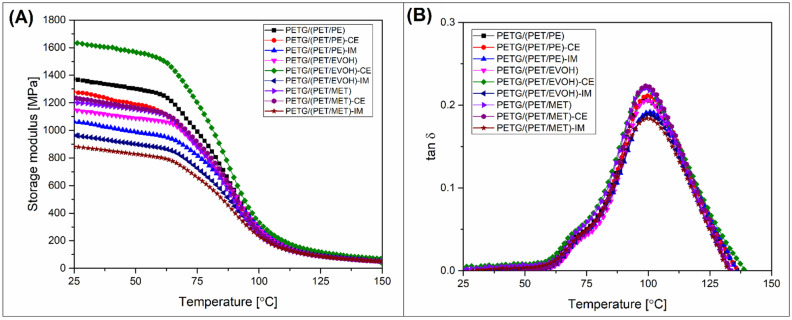
The DMTA results for annealed materials: (**A**) storage modulus; (**B**) tan δ.

**Figure 7:**
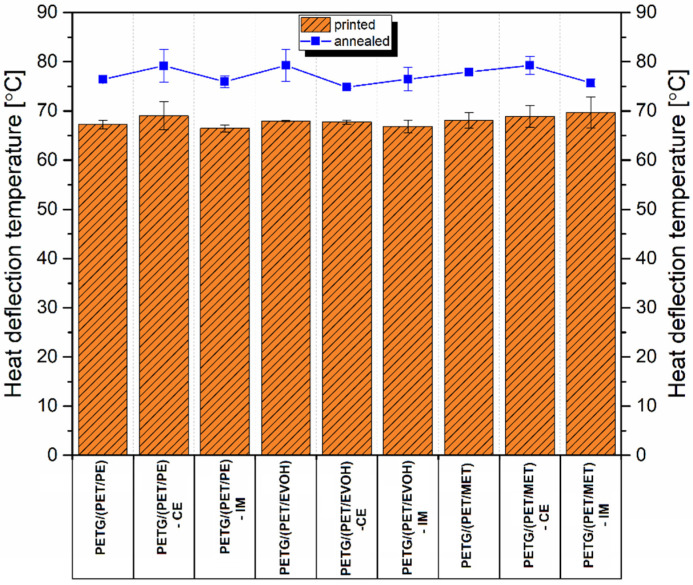
The results of HDT measurements for FDM printed samples and annealed specimens.

The thermomechanical properties of the manufactured materials were investigated using DMTA analysis and HDT testing. The comparison of these two techniques allows us to assess more deeply how the mechanical performance was changing along the temperature scale. Moreover, the complex analysis is helpful to indicate the influence of the annealing procedure on phase transition changes. The results of the DMTA measurement are presented separately for printed samples (see Figure 5) and annealed specimens (Figure 6), while the HDT test results are collected in Figure 7.

The storage modulus results for the printed samples revealed large differences in stiffness even at room temperature. It is clear that for IM-modified samples the results of the storage modulus will strongly correspond to the standard tensile tests, while the 20% content of the POE-g-GMA phase must lead to stiffness deterioration. For the rest of the samples, the visible drop in modulus was recorded for PETG/(PET/MET)-based samples, which had already been observed during the static tests. In the context of thermal resistance, more significant changes begin after the temperature exceeds 60 °C at the PET/PETG glass transition area. Interestingly, the observed drop in storage modulus was a two-stage process, where the observed decrease was relatively slow during the first stage. However, when the temperature reaches around 75 °C, the stiffness drops more rapidly. The storage modulus recorded at 90 °C was below 20 MPa for all of the investigated samples, which transfers into a large softening tendency. The material deflection was observed after the test, where samples were slightly deformed.

The tan δ plots reveal the same behavior since the initial drop in the stiffness was marked by a small peak around 70 °C. The main T_g_ transition was manifested by a large peak at around 85 °C. The direct analysis of the T_g_ area results would indicate the presence of a clearly two-phase structure, probably an immiscible polymer system. However, in practice, PET/PETG-based systems are usually miscible. Usually, the glass transition for polymer blends with good miscibility is manifested with a single tan δ peak [82,83]. Additionally, the content ratio of both polymers is very similar, which would result in a similar area size under the first and second peaks. The observed nature of the T_g_ transition area is difficult to interpret since PETG and PETG/PET-based blends are the subject of research of only a few studies [84,85,86]. Due to the rather complex nature of the used blend system in the described results, it is difficult to rely on the results of other works. Interestingly, similar results indicating the presence of a small initial tan δ peak were recorded for PET nanocomposites [87]; however, the authors did not discuss this phenomenon. Taking into account the results of the DMTA analysis for annealed samples, where the inflection point at the main tan δ peak was recorded in the same area close to 75 °C, the observed bimodal nature of the glass transition can be connected with the presence of a large amount of low molecular weight PET chains. 

The heating procedure was continued up to 150 °C, with the same heating rate. The plateau area observed after exceeding the PET glass transition range was not uniform since the storage modulus values slightly increased before reaching the final test temperature. That change correlates with the DSC test result and is connected with the cold crystallization phenomenon. The improvement in stiffness is the natural consequence of the increasing content of the PET crystalline phase. The observed behavior indirectly confirms the correct choice of the annealing process temperature of 150 °C, while it confirms that the heat treatment process is closely related to the change in thermomechanical properties.

As expected, the storage modulus analysis results for annealed samples revealed large changes in heat resistance. For all of the investigated samples the rapid drop of stiffness around the T_g_ area was not observed; instead, the storage modulus gradually decreased its value, but even for the final temperature (150 °C) the results indicated a modulus of around 150 MPa, which confirmed the high thermal stability above the glass transition region.

The results of the thermomechanical properties evaluation are presented as a comparison between the FDM printed samples and printed/annealed ones. Figure 7 reveals the HDT test results; the test was performed using a 1.8 MPa load, while usually, for 3D printed parts, a 0.455 MPa load is used. As we are expecting to use the developed materials in technical applications, we decided to change the test conditions. 

As confirmed by other research studies, the utilization of a semi-crystalline component is expected to raise the thermal resistance of the prepared blends; the results of the tests for FDM printed samples did not show any improvement compared to unmodified resin [88]. The HDT for the unmodified PETG sample reached 68 °C, while the results for prepared blends ranged from 66 °C to 70 °C. This lack of a noticeable difference suggests that the structure of the manufactured samples was still mostly amorphous despite the presence of the PET component. The obvious reason for that is the relatively slow crystallization kinetic for PET resin. The cooling condition of the FDM printing process ensures rapid cooling of the polymer melt below the crystallization region, which means that both blend components remain amorphous. It is worth adding that, for the obtained blend systems where both polymer species have excellent mutual miscibility, obtaining a high degree of crystallinity is extremely difficult, which is due to the fact that the mobility of PET homopolymer macromolecules is limited by entanglement with PETG copolymer chains. In other cases, when the miscibility of polymers is limited, and one of the phases of the system forms a separated phase or inclusion, the mobility of the macromolecular structure is similar to that occurring in an unmodified polymer. However, the properties of the material are then determined by a simple rule of mixtures. When the semicrystalline polymer content does not exceed 50% of the volume content, obtaining a significant improvement in thermomechanical properties is extremely difficult. The changes observed for the annealed samples are somehow surprising since the DMTA plots reveal a visible improvement in stiffness at higher temperatures. Instead of a large increase, the HDT values are only slightly improved, ranging from 75 °C to 79 °C. That translates into an average improvement of 10 °C. Compared to other research studies involving an annealing procedure, the obtained thermal resistance improvement should be considered small [58,89]. It is worth noting that for the prepared materials the amorphous PETG component constituted approximately half of the mass of the composition, which significantly limited the impact of the increase in crystallinity for the PET phase.

### 3.4. Scanning Electron Microscopy Analysis (SEM)—The Evaluation of the Structure Differences

**Figure 8:**
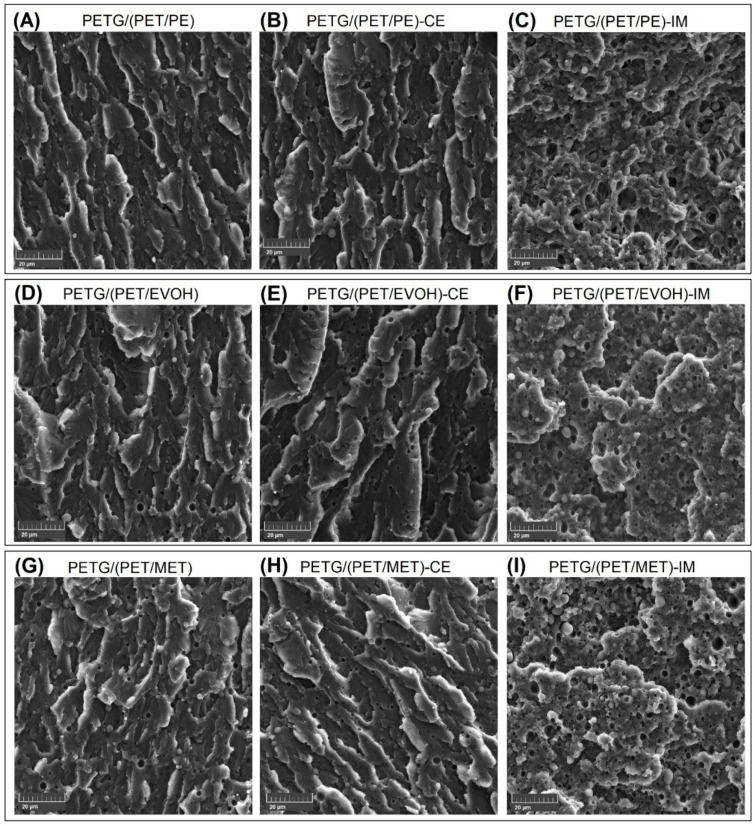
The scanning electron microscopy analysis for injection molded specimens: The scanned surface was obtained from cryo-fractured samples.

The structure of the manufactured materials was investigated using the SEM analysis method. For this purpose, a cryo-fractured sample prepared by the injection molding method was utilized as the investigated specimen. The 3D printed specimens were not suitable for this purpose since most of the samples broke into small pieces during the breaking process, which made positioning in the microscope chamber difficult. The SEM micrographs pictures presented in Figure 8 were taken at 2 k magnification. 

It is clear that for the investigated specimens the main matrix component was the PETG/PET blend. Since the PET-based films contained approximately several percent of PE and/or PE/EVOH, the PETG component had the largest share in the mixture. Interestingly, the structural analysis does not indicate the separation of separate phases of PET and PETG, which suggests at least partial miscibility of these two polymers. The miscibility of PET with other types of copolymers is a confirmed behavior [86,90,91]. Still, the kinetics of this process sometimes depend on the molecular weight or the method of forming the crystalline phase of the individual phases of the mixture [92,93]. Despite the lack of elastomer (IM) addition, the fracture structures for the remaining samples indicate the presence of small amounts of inclusions of the immiscible phase. There are some differences in the size and quantity of these particles for individual samples, but, even without careful image analysis, the content of these inclusions reaches several percent. Inclusions of this type obviously come from the LLDPE fraction present in each of the films used during the preparation of the blend. Similar behavior was observed by Vaucher et al. [64]; however; for the cited research, the addition of HDPE (high-density polyethylene) was used intentionally, while, for the described materials, LLDPE appeared as one of the foil layers; therefore, its presence in the blend was rather undesirable but impossible to avoid. Despite the relatively small amount of PE inclusions, the presence of these inclusions certainly negatively affects the mechanical properties.

The analysis of the structure for materials with the addition of POE-g-GMA elastomer revealed a very similar appearance for each of the manufactured blends. The size of the inclusions ranged from 1 to 8 μm, which is a typical value for this kind of additive [94,95,96]. Structural analysis indirectly confirms the results of mechanical tests, which clearly indicate a more favorable deformation mechanism for materials with the addition of an elastomer fraction. A natural suggestion for further work on this topic could be to optimize the composition of the developed blends, especially in terms of the impact modifier (IM) content. The constant content of 20% used during the tests was estimated in accordance with the guidelines of manufacturers of this type of additive, as well as previous research in this area, including our own [53,97,98]. The use of a smaller amount of the IM additive could maintain the higher stiffness/strength of the prepared blends; additionally, the reduced content of the relatively expensive elastomer would also be economically justified.

### 3.5. Rheological Analysis of the Prepared Materials, Melt Flow Rate Evaluation

**Figure 9:**
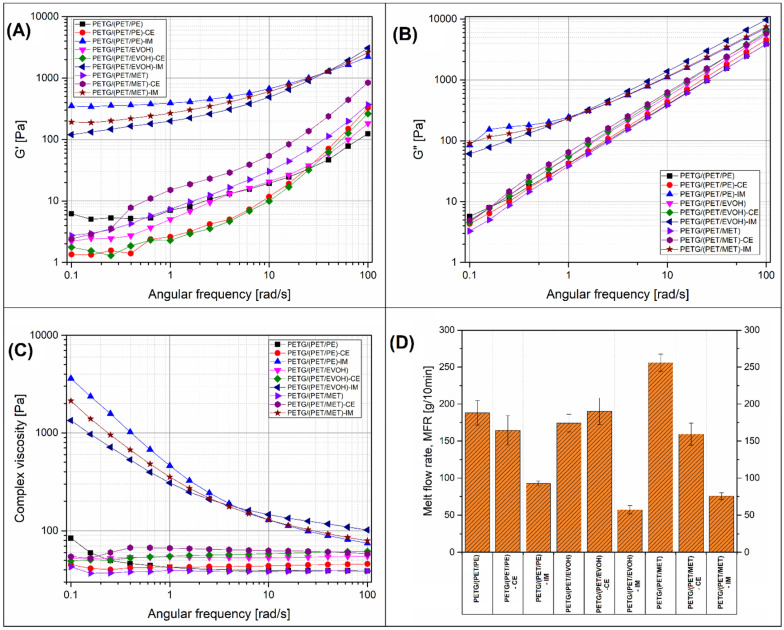
The results of the small-amplitude oscillatory shear tests: (**A**) G′ storage modulus; (**B**) G″ loss modulus; (**C**) complex viscosity. (**D**) Melt flow rate test (MFR) results.

The rheological analysis was conducted using two types of testing methods. The rotational rheometer was used to conduct the small-amplitude oscillatory shear test (SAOS), while the MFR test was conducted in order to compare the viscosity differences in the standard melt flow test. Both types of measurements were conducted at 250 °C. The results of the SAOS measurements are presented in the form of G′/G″ modulus and complex viscosity plots together with the MFR test results, and collected in Figure 9.

**Figure 10:**
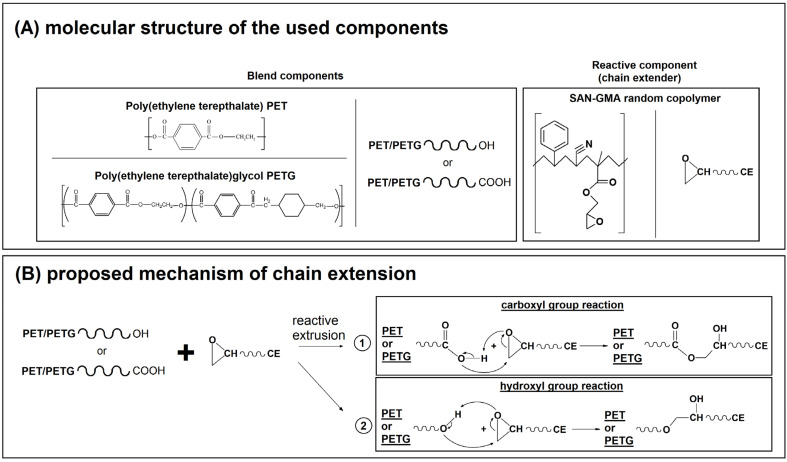
A scheme presenting (**A**) the structure of the main components of the reactive extrusion process and (**B**) the proposed reaction mechanism for the prepared blends.

The results of the tests performed using a rotational rheometer clearly revealed the large difference between the rubber-toughened materials and the rest of the prepared blends. For both G′ and G″ modulus, the plot position for IM-modified samples is strongly shifted to higher modulus values, which is definitely connected with the presence of an elastomeric phase. The increase in elastic tendencies in the material with the addition of IM is reflected in the course of the complex viscosity curves, where, again, the properties of the blends with the addition of POE-g-GMA copolymer differ from other materials. In the case of other materials, including those with the addition of CE, the increasing tendency is not observed. The observed behavior indicates the complex nature of phase interactions during the reactive extrusion process. The diagram shown in Figure 10 presents the possible mechanism of chemical reactions that may occur during the reactive extrusion process. In the case of both main components of the mixture, the presence of carboxyl or hydroxyl groups at the ends of polymer chains is possible. In the case of both main components of the mixture, it is possible to have carboxyl or hydroxyl groups at the ends of polymer chains, and these are the bonding points with reactive components; for the used CE system, these are epoxy groups. In the case of a chemical reaction using polymer chain extenders, the connection with the reactive group in the functionalized oligomer used increases the molecular weight of the polymer. In other cases, it is possible to combine two different macromolecules with a common bond, resulting in the formation of a copolymer. This type of interaction increases the level of compatibilization of polymer blends. For the described toughened blend system, it is possible that there may be additional reaction mechanisms between the PET end groups and the functional groups present in the elastomer chain structure. However, in principle, due to the significantly higher molecular weight of the POE-g-GMA copolymers, this mechanism has much lower kinetics compared to the used chain extender. It is possible that changes in viscosity for blends with the addition of the IM phase are partially related to the occurrence of an effective chemical reaction between the elastomer inclusions and the main PETG/PET blend.

Due to the different compositions, the viscosity charts of unmodified mixtures may differ from each other, but, in principle, the addition of chain extenders is intended to increase the molecular weight and improve the viscosity. However, it is worth mentioning that the intensity of the reactive extrusion process depends on many factors, both material and process, often interdependent. Therefore, the obtained results indicate the lack of direct correlation between viscosity and CE addition, but this does not exclude the occurrence of correlations at the phase interface, where the processes of formation of new polymer systems, including copolymers, may affect the structural properties in the solid phase. Similarly to the SAOS rheological analysis, the results of the MFR measurements indicate a large decrease in material flowability for IM-modified blends. Apart from the obvious fact of an increase in viscosity for materials with the addition of IM-elastomer, the expected effect was a decrease in the MFR index for blends with the addition of CE. Interestingly, this trend is true for only part of the materials, the PETG/(PET/PE)-CE and PETG/(PET/MET)-CE samples, while the melt flow index for the PETG/(PET/EVOH)-CE blend was slightly higher than the unmodified polymer mixture. The most evident differences in viscosity were recorded for the PETG/(PET/MET) samples, where the initial MFR value of around 260 g/10 min was reduced to 160 g/10 min for CE-modified materials, and to 75 g/10 min after addition of elastomer (IM). The results of the viscosity measurements analyzed constitute an important processing indicator for the developed polymer blends and confirm observations made during the extrusion of filament samples. Unlike the injection molding processing, where low viscosity usually positively affects the quality of manufactured products, the FDM filament production requires materials with a lower melt flow rate [99,100]. It is difficult to clearly indicate what value of the viscosity parameter or MFR index is optimal, hence the lack of guidelines or recommendations on this issue. However, from a technological point of view, it is much more favorable to use a material with a moderate or higher viscosity, where an increasing temperature of processing can be used to improve the flow intensity. This allows for a simple and intuitive way to optimize the melt processing conditions for filament extrusion and 3D printing. For this reason, for the developed blends with the addition of foil waste, the most favorable rheological properties occur for samples with the addition of an IM phase, regardless of the initial polymer blend.

## 4. Conclusions

The results of the performed study indicate that the development of valuable waste polymer-based materials is possible for the FDM printing process. The results of the mechanical performance analysis revealed that the difference between the injection molded and FDM printed samples can be significant, usually in favor of molded materials. However, unfavorable trends usually occur for unmodified blends; when properly carried out, modification allows for quite good mechanical characteristics that are only slightly lower than those of injection-molded samples. Interestingly, even for such a critical parameter as impact resistance, it is possible to obtain a relatively high strength value in Charpie tests. For molded samples it is a value of about 15 kJ/m^2^, while for the printed counterparts it is possible to obtain values of about 8 kJ/m^2^. Interestingly, very similar results were obtained for all blends in HDT thermomechanical tests, which clearly confirms that the significant addition of PETG copolymer (40–50%) determines the homogeneity of this parameter. Even the additional annealing treatment leads to a relatively small thermal resistance improvement of around 10 °C. Additionally, the annealing procedure leads to a large deterioration in the mechanical characteristics, which suggests that the developed procedure should be performed for other types of blends. An important benefit resulting from the addition of the POE-g-GMA elastomer phase is the ability to increase the viscosity of the polymer blends. In the case of the FDM printing method discussed in this paper, the ability to control the flow parameters turns out to be very important, especially at the stage of preparing the semi-finished product in the form of a filament. For the processed materials, the addition of a waste PET fraction results in an increase in the MFR index even above 260 g/10 min, which significantly limits the ability to control the shape of the extruded filament as well as the path of the material extruded through the printer nozzle. The obtained results suggest high application potential for the prepared materials. As part of future work, a series of tests are planned to assess the possibility of further modification of the presented blends using composite additives.

## Figures and Tables

**Table 1:** The list of prepared samples and blend composition (presented as wt%).

Sample	PETG	Foil 1 (PET/PE)	Foil 2 (PET/EVOH)	Foil 3 (PET/MET)	Chain Extender (CE)	Impact Modifier (IM)
PETG (pure)	100	-	-	-	-	-
PETG/(PET/PE)	50	50	-	-	-	-
PETG/(PET/PE)-CE	50	49.5	-	-	0.5	-
PETG/(PET/PE)-IM	40	39.5	-	-	0.5	20
PETG/(PET/EVOH)	50	-	50	-	-	-
PETG/(PET/EVOH)-CE	69	-	49.5	-	0.5	
PETG/(PET/EVOH)-IM	40	-	39.5	-	0.5	20
PETG/PET/MET	50	-	-	50	-	-
PETG/PET/MET-CE	50	-	-	49.5	0.5	-
PETG/PET/MET-IM	40	-	-	39.5	0.5	20

**Table 2:** A compilation of the mechanical properties of samples prepared by the FDM method; the reported values are collected from the literature.

Blend Type	Mechanical Properties	References
LDPE/PET/Aluminum	Tensile strength: 10–14 MPa Elongation: 4–13%	[10]
rPET/rHDPE	E modulus: 1.7–2.9 GPa Tensile strength: 32–65 MPa	[64]
rPE/rPET	E modulus: 65–90 GPa Tensile strength: 7–23 MPa	[65]
rPET/rHDPE	E modulus: 0.8–1.6 GPa Tensile strength: 12–31 MPa Elongation: 1.6–5.3%	[66]
PLA/recycled glass fiber	E modulus: 3.2 GPa Tensile strength: 50 MPa	[67]
PLA reprocessing	E modulus: 3.5–5.5 GPa Tensile strength: 27–39 MPa Elongation: 3.7–4.9%	[68]
PLA reprocessing	E modulus: 3.2–3.4 GPa Tensile strength: 52–62 MPa Elongation: 2.0–2.7%	[69]
This study	E modulus: 1.4–2.7 GPa Tensile strength: 27–53 MPa Elongation: 2.0–50%	

**Table 3:** The basic thermal properties collected from the DSC plots.

Material	Printed Materials				Printed/Annealed Materials			
	T_g_	T_cc_	T_m_	T_c_	T_g_	T_m′_	T_m_	T_c_
PETG/(PET/PE)	74.2	146.2	241.8	-	80.8	159.2	239.9	-
PETG/(PET/PE)-CE	74.9	145.6	243.0	151.1	81.2	158.9	237.9	-
PETG/(PET/PE)-IM	74.1	138.2	247.0	158.5	80.5	158.0	243.5	158.3
PETG/(PET/EVOH)	74.3	146.6	242.4	-	80.4	156.8	242.9	-
PETG/(PET/EVOH)-CE	74.1	145.1	242.2	-	80.9	157.8	241.8	-
PETG/(PET/EVOH)-IM	73.9	139.9	245.6	150.4	81.3	152.2	244.2	151.4
PETG/PET/MET	75.6	145.4	240.5	97.0 *	81.0	156.2	236.6	94.8 *
PETG/PET/MET-CE	74.9	145.6	243.0	151.1	80.8	157.6	239.8	96.2 *
PETG/PET/MET-IM	74.1	138.2	247.0	158.5	80.7	159.9	244.8	157.7/92.1 *

* The T_c_ related with LLDPE content.

## Data Availability

The data presented in this study are available on request from the corresponding author due to privacy.
